# Miltefosine Failure and Amphotericin B Success in the Treatment of a Case of Cutaneous *Leishmania Braziliensis* in a Recent Traveler in Belize and Guatemala

**DOI:** 10.1155/crdi/6644758

**Published:** 2025-07-05

**Authors:** Michelle Y. Ko, Emilie Fowler, Amanda Scott, Daniel Z. Uslan

**Affiliations:** ^1^David Geffen School of Medicine at the University of California, Los Angeles, California, USA; ^2^Division of Dermatology, David Geffen School of Medicine at the University of California, Los Angeles, California, USA; ^3^Santa Barbara Lifestyle Medicine, Santa Barbara, California, USA; ^4^Division of Infectious Disease at the David Geffen School of Medicine at the University of California, Los Angeles, California, USA

**Keywords:** amphotericin B, Belize, cutaneous leishmaniasis, Guatemala, miltefosine

## Abstract

We report the case of a 53-year-old male with recent travel to Guatemala and Belize who was diagnosed with cutaneous leishmaniasis (CL). He was treated empirically with miltefosine with no improvement and switched to amphotericin B upon species identification of *L. braziliensis*, resulting in the resolution of his lesions. This case demonstrates that clinicians should recognize the importance of systemic therapy for treating complex CL, as well as the importance of identification of species type for tailoring treatments. Furthermore, while miltefosine has proven efficacious for CL in many New World locales, it has demonstrated lower cure rates for CL in Guatemala, and thus identification of the region of origin of the CL infection is imperative for further guiding treatment, which may vary according to the differences in drug potency or region-specific resistance rates.

## 1. Background

Leishmaniasis is a vector-borne infection caused by the protozoan parasite *Leishmania* and transmitted by the female sandfly [1]. Clinical infections may be categorized into three main syndromes: cutaneous leishmaniasis (CL) characterized by cutaneous ulcers, mucocutaneous leishmaniasis (MCL) characterized by mucosal and cartilaginous involvement, and visceral leishmaniasis (VL), also called kala-azar leishmaniasis, characterized by internal organ involvement [2]. The manifestation of the disease depends on the *Leishmania* species, which is largely categorized into those found in the Old World, referring to the Mediterranean, the Middle East, Africa, and India, and those found in the New World, referring to the Americas [1]. As infections caused by different *Leishmania* species often require different treatment approaches, it is critical to identify *Leishmania* species for optimal management. Herein, we report the case of a 53-year-old male with recent travel to Guatemala and Belize who was diagnosed with CL, treated empirically with miltefosine with no improvement, and then switched to amphotericin B upon species identification of *L. braziliensis*, resulting in the resolution of his lesions.

## 2. Case Presentation

**Figure 1:**
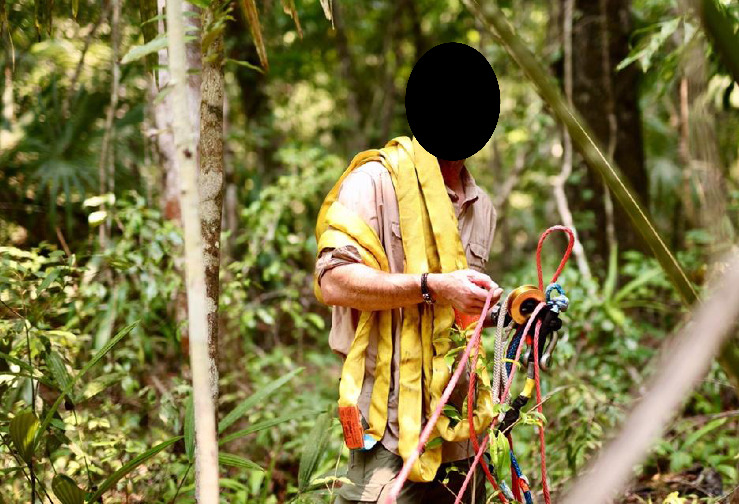
The patient would like to share the following image as a representation of the environment of the patient's travels during his expedition. The patient believes he was bitten around the time the following photo was taken when he was hooking up a line to move trees out of their tracks.

**Figure 2:**
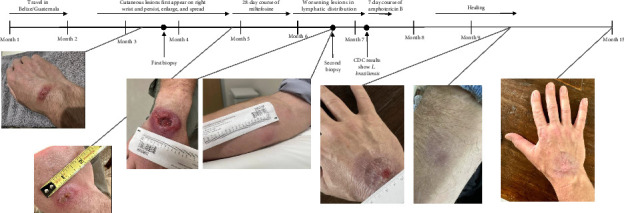
Timeline of patient's cutaneous lesions and medications, format adapted from Joseph et al. 2021 [3].

A 53-year-old male previously in good health traveled to Belize and Guatemala for one month. His trip involved several rigorous hikes through rural jungles and included camping overnight in remote areas with minimal shelter (Figure 1). He endorsed multiple insect and spider bites. After returning to the United States 2 weeks later, he noticed a new small lesion on the right wrist. Clinical examination revealed a 0.5 cm circular, raised, erythematous papule with central ulceration on the dorsal aspect of the right wrist (Figure 2). The patient denied fevers, congestion, rhinorrhea, and sore throat. The patient endorsed weight loss and fatigue, although he attributed these symptoms to his trip. Repeat clinical examination 1 month later revealed a large, 4.5 cm erythematous plaque with central ulceration on the dorsal aspect of the right wrist and several new, tender, subcutaneous nodules with linear extension along a lymphatic distribution on the right forearm.

**Figure 3:**
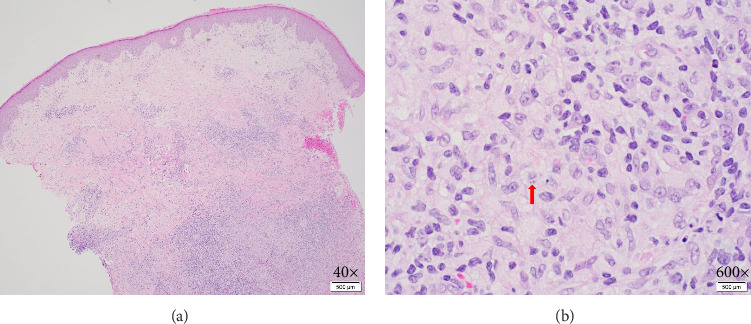
Punch biopsy of the right forearm taken after miltefosine treatment completion showing (a) dense, dermal, lymphohistiocytic inflammation with scattered granulomas and foci of necrosis suggestive of an infectious process. (b) Rare structures within histiocytes (red arrow), likely degenerated amastigotes given the patient's history of treated cutaneous leishmaniasis.

Biopsy at an outside institution revealed small hematoxylinophilic, round, uniform intracytoplasmic amastigotes distributed around the outer rim of vacuoles (marquee sign) within histiocyte–macrophage components, morphologically suggestive of *Leishmania* amastigotes (Figure 3). The biopsy was formalin-fixed and paraffin-embedded (FFPE). The specimen was sent to the Centers for Disease Control and Prevention (CDC), where *Leishmania* was confirmed, but species ID could not be performed on the FFPE tissue, so the patient was recommended to return to the clinic and undergo a second biopsy. Videostroboscopy and nasal endoscopy demonstrated no nasal or upper aerodigestive lesions.

The patient was diagnosed with CL and treated empirically with a 28-day course of miltefosine at 2.5 mg/kg/day while awaiting a second biopsy to be sent to the CDC for *Leishmania* species identification. Although the patient experienced mild nausea with the first few doses of miltefosine, he was able to tolerate the medication and complete the total course as prescribed. Six weeks after completing the miltefosine course, the patient failed to demonstrate improvement. At that time, results from the CDC from the second biopsy returned, identifying *Leishmania braziliensis* as the causative species. The patient was then started on liposomal amphotericin B (AmBisome®) at 3 mg/kg for 7 days (cumulative dose of 1858.5 mg), which he tolerated well with some fatigue and nausea and transiently raised creatinine levels that resolved. Eventually, the patient experienced resolution of the lesions and upon follow-up 2 months later had not experienced recurrence of any lesions.

## 3. Discussion and Conclusions

Management of CL depends on the *Leishmania* species, which is often predicted by the region of origin. According to recent clinical guidelines, patients may be categorized as having simple or complex CL. Patients are categorized as having complex CL if they have any of the following: *Leishmania* species typically associated with MCL; greater than 4 lesions that are greater than 1 cm in diameter; individual lesion greater than or equal to 5 cm; local subcutaneous nodules or large regional lymph nodes; lesions on the face, digits, joints, or genitalia; immunocompromised status; or failure of treatment [4, 5]. Those with simple CL with lesions that have not yet begun to heal may be treated with local therapies, whereas those with simple CL with lesions that have already begun to heal may be observed [4, 5]. Those with complex CL should be treated with systemic therapy. Our patient satisfied several of the criteria for being categorized as complex CL, including single lesions eventually growing to greater than or equal to 5 cm, involvement of lymphatic nodules, and failure of treatment.

Given that speciation of leishmaniasis cultures requires outsourcing to a limited number of reference laboratories and thus take time to result, the decision was made to empirically treat the patient with systemic treatment with miltefosine [4, 5]. Our patient acquired the disease in either Belize or Guatemala, which border each other. In Guatemala, several studies conducted in the 1980s and 1990s revealed that most CL lesions (73%–75%) were due to *L. braziliensis*, with the next most prevalent species being *L. mexicana*. Although L. *braziliensis* is commonly associated with MCL, MCL has rarely been reported in Guatemala [6]. The data on *Leishmania* in Belize are more sparse. One Belize CL study on British troops touring in Belize in the early 1990s found that in 306 soldiers with a clinical diagnosis of CL, 187 cases had parasitological confirmation, of which, 78 were identified as *L. braziliensis* and 29 as *L. mexicana*, and none with MCL [7]. There have also been a handful of published case reports of leishmaniasis originating from Belize, with all caused by either *L. braziliensis* or *L. mexicana.* [8–10].

Miltefosine (Impavido®) is the first and only oral drug approved by the United States' Food and Drug Administration (FDA) to treat CL, including that caused by *L. braziliensis* [11]. It is an alkylphosphocholine drug that is generally well tolerated, with the exception of gastrointestinal side effects, such as nausea and vomiting [11]. The endemic region in which the infection is acquired, however, affects miltefosine's efficacy. Although Bahia, Brazil, and Guatemala are both regions in which *L. braziliensis* predominate, the cure rate for CL with miltefosine was 85% in Bahia, Brazil, yet only 48% in Guatemala [12]. The data regarding miltefosine efficacy for CL in Belize are even more limited. Interestingly, one case report described two cases of CL due to *L. braziliensis* in Dutch military personnel acquired in Belize that were treated successfully with oral miltefosine [13].

Given that our patient experienced worsening lesions despite completing treatment with miltefosine, it is possible that our patient may have acquired the infection in Guatemala rather than Belize, though it is impossible to confirm. Given the progression of disease after completion of miltefosine therapy, the patient was started on amphotericin B. Amphotericin B is a polyene drug that has been approved by the FDA to treat VL, though it has been used for complex CL as well. Although highly effective, amphotericin B carries several severe side effects including nephrotoxicity, which may be offset by saline loading in patients prior to dosing. The liposomal amphotericin form is often preferred due to its less severe side effect profile. After completing treatment with amphotericin B, our patient experienced subsequent healing of his lesions. He did have a transient creatinine increase to 1.76. With hydration, this normalized to his baseline creatinine of 1.10.

In conclusion, clinicians should recognize the importance of systemic therapy for treating complex CL and of the identification of species type and region of acquisition for tailoring treatments for CL. Furthermore, while miltefosine has proven efficacious for CL in Colombia, it has demonstrated lower cure rates for CL in Guatemala. Thus, identification of the region of origin of the CL infection is imperative for further guiding treatment, which may vary according to differences in drug potency or region-specific resistance rates [14–17].

## Data Availability

The authors have nothing to report.

## Nomenclature

CL: Cutaneous leishmaniasis
MCL: Mucocutaneous leishmaniasis
VL: Visceral leishmaniasis

## References

[1] de Vries H. J. C., Schallig H. D. (2022). Cutaneous Leishmaniasis: A 2022 Updated Narrative Review into Diagnosis and Management Developments. *American Journal of Clinical Dermatology*.

[2] Pradhan S., Schwartz R. A., Patil A., Grabbe S., Goldust M. (2022). Treatment Options for Leishmaniasis. *Clinical and Experimental Dermatology*.

[3] Joseph S., Whitman T. J., Buckner F. S., Cogen A. L. (2021). Case Report: Miltefosine Failure and Spontaneous Resolution of Cutaneous Leishmaniasis Braziliensis. *The American Journal of Tropical Medicine and Hygiene*.

[4] Aronson N. E., Joya C. A. (2019). Cutaneous Leishmaniasis: Updates in Diagnosis and Management. *Infectious Disease Clinics of North America*.

[5] Aronson N., Herwaldt B. L., Libman M. (2016). Diagnosis and Treatment of Leishmaniasis: Clinical Practice Guidelines by the Infectious Diseases Society of America (IDSA) and the American Society of Tropical Medicine and Hygiene (ASTMH). *Clinical Infectious Diseases*.

[6] Lopez Y., Arana B., Rizzo N., Duran E., Acosta-Serrano Á., Mendizabal-Cabrera R. (2023). A Neglected Among the Neglected: a Review of Cutaneous Leishmaniasis in Guatemala. *Transactions of the Royal Society of Tropical Medicine and Hygiene*.

[7] Hepburn N. C., Tidman M. J., Hunter J. A. (1993). Cutaneous Leishmaniasis in British Troops from Belize. *British Journal of Dermatology*.

[8] Demers E., Forrest D. M., Weichert G. E. (2013). Cutaneous Leishmaniasis in a Returning Traveller. *Canadian Medical Association Journal*.

[9] Pollack K., Flowers R., Zlotoff B. (2019). Cutaneous Leishmaniasis in a Boy from Belize. *The Journal of Pediatrics*.

[10] van Thiel P. P. A. M., Zeegelaar J. E., van Gool T., Faber W. R., Kager P. A. (2011). Cutaneous Leishmaniasis in Three Dutch Military Cohorts Following Jungle Training in Belize. *Travel Medicine and Infectious Disease*.

[11] Ware J. M., O’Connell E. M., Brown T. (2021). Efficacy and Tolerability of Miltefosine in the Treatment of Cutaneous Leishmaniasis. *Clinical Infectious Diseases: An Official Publication of the Infectious Diseases Society of America*.

[12] Knight (2025). *U.S. Food and Drug Administration Website*.

[13] van der Snoek E. M., Couwenberg S. M., Stijnis C., Kortbeek L. M., Schadd E. M. (2017). Two Cases of Cutaneous Leishmaniasis in Dutch Military Personnel Treated with Oral Miltefosine. *Journal of the Royal Army Medical Corps*.

[14] Kevric I., Cappel M. A., Keeling J. H. (2015). New World and Old World Leishmania Infections: A Practical Review. *Dermatologic Clinics*.

[15] Sundar S., Olliaro P. L. (2007). Miltefosine in the Treatment of Leishmaniasis: Clinical Evidence for Informed Clinical Risk Management. *Therapeutics and Clinical Risk Management*.

[16] Soto J., Arana B. A., Toledo J. (2004). Miltefosine for New World Cutaneous Leishmaniasis. *Clinical Infectious Diseases*.

[17] Soto J., Berman J. (2006). Treatment of New World Cutaneous Leishmaniasis with Miltefosine. *Transactions of the Royal Society of Tropical Medicine and Hygiene*.

